# Supplementary material for: Sox2 levels regulate the chromatin occupancy of WNT mediators in epiblast progenitors responsible for vertebrate body formation
Source: Nat Cell Biol. 2022 May 12;24(5):633–44. doi: 10.1038/s41556-022-00910-2 (PMC9106585; doi:10.1038/s41556-022-00910-2)
Supplement: Supplementary file 1 — Reporting Summary [file 41556_2022_910_MOESM1_ESM.pdf]

nature research

Corresponding author(s): James Briscoe

Last updated by author(s): 2022 03 01

Reporting Summary

Nature Research wishes to improve the reproducibility of the work that we publish. This form provides structure for consistency and transparency in reporting. For further information on Nature Research policies, see our Editorial Policies and the Editorial Policy Checklist.

Statistics

For all statistical analyses, confirm that the following items are present in the figure legend, table legend, main text, or Methods section.

n/a	Confirmed	
☐	☒	The exact sample size (*n*) for each experimental group/condition, given as a discrete number and unit of measurement
☒	☐	A statement on whether measurements were taken from distinct samples or whether the same sample was measured repeatedly
☐	☒	The statistical test(s) used AND whether they are one- or two-sided<br>*Only common tests should be described solely by name; describe more complex techniques in the Methods section.*
☒	☐	A description of all covariates tested
☒	☐	A description of any assumptions or corrections, such as tests of normality and adjustment for multiple comparisons
☐	☒	A full description of the statistical parameters including central tendency (e.g. means) or other basic estimates (e.g. regression coefficient) AND variation (e.g. standard deviation) or associated estimates of uncertainty (e.g. confidence intervals)
☐	☒	For null hypothesis testing, the test statistic (e.g. *F*, *t*, *r*) with confidence intervals, effect sizes, degrees of freedom and *P* value noted<br>*Give P values as exact values whenever suitable.*
☒	☐	For Bayesian analysis, information on the choice of priors and Markov chain Monte Carlo settings
☒	☐	For hierarchical and complex designs, identification of the appropriate level for tests and full reporting of outcomes
☒	☐	Estimates of effect sizes (e.g. Cohen's *d*, Pearson's *r*), indicating how they were calculated

Our web collection on statistics for biologists contains articles on many of the points above.

Software and code

Policy information about availability of computer code

Data collection

Flow cytometry data was acquired using BD FACSDiva software. RT-PCR data was acquired using Applied Biosystems QuantStudio software. Immunofluorescence images were acquired using Zeiss Zen software.

Data analysis

A full list of all publicly available software used to analyse data is detailed in supplementary table 2 and below:

Applied Biosystems QuantStudio 12K Flex N/A
BAMTools (v2.5.1) Barnett et al., 2011
BD FACS DIVA (Version 8.0.1) N/A
bedGraphToBigWig (v1.0) Kent et al., 2010
BEDTools (v2.27.1) Quinlan and Hall, 2010
BWA (v0.7.17-r1188) Li and Durbin, 2009
cutadapt (version 1.16) Martin, 2011
DAVID (version 6.8) https://david.ncifcrf.gov/
deepTools (v3.2.1) Ramírez et al., 2016
DESeq2 (v1.20.0) Love et al., 2014
FastQC (v0.11.8) https://www.bioinformatics.babraham.ac.uk/projects/fastqc/
featureCounts (v1.6.4) Liao et al., 2014
Fiji (v2.0.0) Schindelin et. al. 2012
FIMO (v5.1.1) http://meme-suite.org/tools/fimo
flowcore (v1.44.2) Ellis et al., 2018
ggplot2 (3.3.0) Wickham, 2016
gplots (v3.1.0) Gregory et. al. 2015
HOMER (v4.10) Heinz et al., 2010
IGV genome browser (v2.4.13) Robinson et al., 2011

MACS (v2.1.2) Zhang et al., 2008
MultiQC (v1.7) Ewels et al., 2016
Nextflow (version 19.10.0) Di Tommaso et al., 2017
NucleoATAC (v0.3.4) Schep et al., 2015
phantompeakqualtools (v1.14) Landt et al., 2012
picard-tools (v2.19.0) http://broadinstitute.github.io/picard
Preseq (v2.0.3) http://smithlabresearch.org/software/preseq/
Prism (Version 8) N/A
Pysam (v0.15.2) https://github.com/pysam-developers/pysam
R (version 3.4.1) R Core Team, 2018
RSEM (version 1.3.0) Li and Dewey, 2011
SAMtools (v1.9) Li et al., 2009
Singularity (version 2.6.0) Kurtzer et al., 2017
STAR (version 2.5.2a) Dobin et al., 2013
Trim Galore! (v0.5.0) https://www.bioinformatics.babraham.ac.uk/projects/trim_galore/

For RNAseq analysis adapter trimming was performed with cutadapt (version 1.16) with parameters "--minimum-length=25 --quality-cutoff=20 -a AGATCGGAAGAGC", and for paired-end data "-A AGATCGGAAGAGC" was appended to the command. The RSEM package (version 1.3.0) in conjunction with the STAR alignment algorithm (version 2.5.2a) was used for the mapping and subsequent gene-level counting of the sequenced reads with respect to mm10 RefSeq genes downloaded from the UCSC Table Browser on 11th December 2017. The parameters passed to the "rsem-calculate-expression" command were "--star --star-gzipped-read-file --star-output-genome-bam --forward-prob 0", and for paired-end data "--paired-end" was appended to the command.

For ChIP-seq analysis the nf-core/ChIP-seq pipeline (version 1.1.0; https://doi.org/10.5281/zenodo.3529400) written in the Nextflow domain specific language (version 19.10.0) was used to perform the primary analysis of the samples in conjunction with Singularity (version 2.6.0). The command used was " nextflow run nf-core/ChIP-seq --input design.csv --genome mm10 --gtf refseq_genes.gtf --single_end --narrow_peak --min_reps_consensus 2 -profile crick -r 1.1.0". To summarise, the pipeline performs adapter trimming (Trim Galore!), read alignment (BWA) and filtering (SAMtools) ; (BEDTools); (BamTools); (pysam); (picard-tools), normalised coverage track generation (BEDTools); (bedGraphToBigWig), peak calling (MACS) (default q-value threshold < 0.05) and annotation relative to gene features (HOMER), consensus peak set creation (BEDTools), and read counting (featureCounts). Inclusion of a peak in the consensus peak set required that it be called by MACS in a minimum of 2/3 biological replicates from any of the four experimental conditions (CEpiLC, SOX2-OFF, SOX2-ON, Naïve ES). In all analyses, except for Fig 3C and Extended Data Fig 4C, the consensus peak set was derived from SOX2 peaks. For Fig 3C and Extended Data Fig 4C the consensus peak set comprised peaks from SOX2/B-catenin/TCF7L1/LEF1. All data was processed relative to the mouse UCSC mm10 genome (UCSC) downloaded from AWS iGenomes (https://github.com/ewels/AWS-iGenomes). Peak annotation was performed relative to the same GTF gene annotation file used for the RNA-seq analysis.

ATAC-seq analysis was performed using the nf-core/atacseq pipeline (version 1.0.0; https://doi.org/10.5281/zenodo.2634133), which uses similar processing steps as described for the nf-core/ChIP-seq pipeline with the addition of the removal of mitochondrial reads.

For nucleosome analysis the NucleoATAC package (version 0.3.4) (Schep et al., 2015) was run in default mode. Analysis was performed on all genomic intervals called as peaks from ATAC-seq data.

bigWig and bedGraph tracks of ChIPseq and NucleoATAC data were visualised using the IGV genome browser to illustrate representative peaks.

Differential analysis of ChIPseq and RNAseq read counts was performed with the DESeq2 package within the R programming environment (version 3.4.1). Log2FC and adjusted p-value of thresholds used for filtering results are indicated in legends.

SOX2 peaks were manually assigned to 6 clusters based upon differential occupancy between WT, 'SOX2 OFF', and 'SOX2 ON' samples. Peaks in clusters 1 ,2 and 3 had the highest mean read counts across biological triplicate samples in either WT, 'SOX2 OFF', or 'SOX2 ON' respectively, and were statistically different (FDR <0.05) as determined by DESeq2 in comparison to all other experimental conditions. Cluster 4, 5, and 6 peaks were statistically different to only one of the other experimental conditions. Bed files of genomic intervals defined by SOX2 peaks within these clusters were used to plot metaplots and heatmaps from the BigWig files generated from the nf-core/ChIP-seq and nf-core/ATAC-seq pipelines using deepTools, for motif enrichment analysis, and for motif scanning.

For GO analysis the online functional annotation tool of the DAVID bioinformatics resource https://david.ncifcrf.gov/summary.jsp was used with default parameters to identify statistically enriched biological process annotations within sets of gene IDs associated with differentially expressed transcripts, and to calculate associated Benjamini-hochberg adjusted p-values.

For motif scanning regions of +/-100bp adjacent to SOX2 ChIP-seq peak centres were used as inputs for the motif scanning tool http://meme-suite.org/tools/fimo. The Sox2 motif MA0143.3 (JASPAR) was used as a target. p-value threshold was set to p <0.1 so as to include low scoring SOX2 motifs present within peak sets.

Motifs enriched within each SOX2 peak cluster were identified using Homer findMotifsGenome using default parameters. Region size = 200b (+/-100bp adjacent to peak centre).

For manuscripts utilizing custom algorithms or software that are central to the research but not yet described in published literature, software must be made available to editors and reviewers. We strongly encourage code deposition in a community repository (e.g. GitHub). See the Nature Research guidelines for submitting code & software for further information.

Data

Policy information about availability of data

All manuscripts must include a data availability statement. This statement should provide the following information, where applicable:

- Accession codes, unique identifiers, or web links for publicly available datasets
- A list of figures that have associated raw data
- A description of any restrictions on data availability

Deep-sequencing (ChIP-seq, ATAC-seq, and RNA–seq) data generated during this study have been deposited in the Gene Expression Omnibus (GEO) under the accession code GSE162774. Previously published ChIP-seq, ATAC-seq, and RNA–seq data that were re-analysed during this study are available under accession codes GSE64059, GSE84899, GSE93524, E-MTAB-2268, E-MTAB-2958, E-MTAB-6337. All other data are available from the authors on reasonable request.

Field-specific reporting

Please select the one below that is the best fit for your research. If you are not sure, read the appropriate sections before making your selection.

☒ Life sciences ☐ Behavioural & social sciences ☐ Ecological, evolutionary & environmental sciences

For a reference copy of the document with all sections, see nature.com/documents/nr-reporting-summary-flat.pdf

Life sciences study design

All studies must disclose on these points even when the disclosure is negative.

Sample size	Sample size of RNAseq and ChIPseq experiments was based on previous experience gained during the studies of Metzis et. al. 2018, Gouti et. al. 2014 & 2017, and other similar studies analysed by the Crick Institute Bioinformatics and Biostatistics group. 3 biological replicates were deemed the minimum necessary to provide sensitivity for detection of differential features by DESeq2 analysis. For qRT-PCR assays all samples collected over the course of exploratory and validatory stages of the study were aggregated for statistical analysis.
Data exclusions	Melt-curve analysis and extreme cT values were used as a basis for exclusion outliers of qPCR technical replicates prior to statistical analysis of data.
Replication	All experimental findings reported were reproducible. Experimental data presented in this study was obtained from a minimum of three independent experiments, each containing one or more biological replicates. This allowed evaluation of both within- and between experiment variability. Multiple RT-PCR and flow cytometry experiments were performed to confirm the robustness of experimental observations before proceeding to perform comprehensive analysis using next-generation sequencing assays.
Randomization	No randomisation was used to allocate cell culture samples to groups when comparisons were made between cell lines of different genotypes. When cell culture samples of the same genotype were compared for their response to a compound or cytokine, multiple samples were plated and assigned randomly to treatment or control groups.
Blinding	Investigators were not blinded during data collection or analysis. Experiments in this study required frequent intervention by the researcher to maintain cell cultures that precluded effective blinding.

Reporting for specific materials, systems and methods

We require information from authors about some types of materials, experimental systems and methods used in many studies. Here, indicate whether each material, system or method listed is relevant to your study. If you are not sure if a list item applies to your research, read the appropriate section before selecting a response.

Materials & experimental systems

n/a	Involved in the study	
☐	☒	Antibodies
☐	☒	Eukaryotic cell lines
☒	☐	Palaeontology and archaeology
☒	☐	Animals and other organisms
☒	☐	Human research participants
☒	☐	Clinical data
☒	☐	Dual use research of concern

Methods

n/a	Involved in the study	
☐	☒	ChIP-seq
☐	☒	Flow cytometry
☒	☐	MRI-based neuroimaging

Antibodies

Antibodies used	All antibodies used in this study are detailed in supplemental table 1 and below:

Mouse OCT3/4 Santa cruz Cat#sc-5279 RRID:AB_628051 1:500
Rabbit NANOG Abcam Cat#ab80892 RRID:AB_2150114 1:500
Goat T/BRA R&D Cat#AF2085 RRID:AB_2200235 1:500
Rabbit SOX2 Millipore Cat#AB5603 RRID:AB_2286686 1:500
Mouse Sox2 V450 BD Cat#561610 RRID:AB_10712763 1:100
Mouse CDX2 647 BD Cat#560395 RRID:AB_1645405 1:20
Mouse CDX2 PE BD Cat#563428 RRID:AB_2738198 1:50
Goat Bra 488 R&D Cat#IC2085G 1:100
Goat SOX2 R&D Cat#AF2018 RRID:AB_355110 Lot#KOY0316101 5ug/IP
Rabbit B-Catenin Invitrogen Cat#71-2700 RRID:AB_2533982 Lot#SH2565754 5ug/IP
Goat TCF3 Santa Cruz Cat#sc-8635 RRID:AB_2199133 Lot#L1415 5ug/IP
Goat TCF4 Santa Cruz Cat#sc-8631 RRID:AB_2199826 Lot#K0615 5ug/IP
Goat LEF1 Millipore Cat#CS200635 Lot#2816642 3ug/IP
Donkey anti-Rabbit IgG AlexaFluor 647, ThermoFisher, Cat#A31573, 1:1000
Donkey anti-Goat IgG AlexaFluor 647, ThermoFisher, Cat#A21447, 1:1000
Donkey anti-Rabbit IgG AlexaFluor 488, ThermoFisher, Cat#A21206, 1:1000
Donkey anti-Mouse IgG AlexaFluor 488, ThermoFisher, Cat#A21202, 1:1000

Validation

Antibodies used in this study are established reagents and have been validated in previous studies. References and links to manufacturers websites are provided below:

Mouse OCT3/4 PMID: 35143761 https://www.scbt.com/p/oct-3-4-antibody-c-10
Rabbit NANOG PMID: 33472064 https://www.abcam.com/nanog-antibody-ab80892
Goat T/BRA PMID: 25157815 https://www.rndsystems.com/products/human-mouse-brachyury-antibody_af2085
Rabbit SOX2 PMID: 25115237 https://www.merckmillipore.com/GB/en/product/Anti-Sox2-Antibody,MM_NF-AB5603
Mouse Sox2 V450 PMID: 34536382 https://www.bdbiosciences.com/en-gb/products/reagents/flow-cytometry-reagents/research-reagents/single-color-antibodies-ruo/v450-mouse-anti-sox2.561610
Mouse CDX2 647 PMID: 34536382 https://www.bdbiosciences.com/en-us/products/reagents/flow-cytometry-reagents/research-reagents/single-color-antibodies-ruo/alexa-fluor-647-mouse-anti-cdx-2.560395
Mouse CDX2 PE PMID: 34536382 https://www.bdbiosciences.com/en-eu/products/reagents/flow-cytometry-reagents/research-reagents/single-color-antibodies-ruo/pe-mouse-anti-human-cdx-2.563428
Mouse Bra 488 PMID: 34536382 https://www.rndsystems.com/products/human-mouse-brachyury-alexa-fluor-488-conjugated-antibody_ic2085g
Goat SOX2 PMID: 28826820 https://www.rndsystems.com/products/human-mouse-rat-sox2-antibody_af2018
B-Catenin PMID: 28826820 https://www.thermofisher.com/antibody/product/beta-Catenin-Antibody-clone-CAT-15-Polyclonal/71-2700
TCF3 PMID: 18347094 https://www.scbt.com/p/tcf-3-antibody-m-20
TCF4 PMID: 24413017 https://www.scbt.com/p/tcf-4-antibody-n-20
LEF1 PMID: 24413017 https://www.merckmillipore.com/GB/en/product/ChIPAb-LEF1-ChIP-Validated-Antibody-and-Primer-Set,MM_NF-17-604

Eukaryotic cell lines

Policy information about cell lines

Cell line source(s)

The embryonic stem cell line, HM1 TetON, used in this study was first described in Serafimidis et. al. and were a gift from Anthony Gavalas to James Briscoe.

Authentication

Karyotyping was performed to verify genome integrity.

Mycoplasma contamination

All cells used in this study were routinely tested for Mycoplasma. No mycoplasma infected cells were used in this study.

Commonly misidentified lines
(See ICLAC register)

No lines from this register were used in the study.

ChIP-seq

Data deposition

☒ Confirm that both raw and final processed data have been deposited in a public database such as GEO.

☒ Confirm that you have deposited or provided access to graph files (e.g. BED files) for the called peaks.

Data access links
May remain private before publication.

https://www.ncbi.nlm.nih.gov/geo/query/acc.cgi?acc=GSE162774

Files in database submission

WT_NAIVE_BCATENIN_IP_R1.fastq.gz
WT_NAIVE_BCATENIN_IP_R2.fastq.gz
WT_NAIVE_BCATENIN_IP_R3.fastq.gz
WT_NAIVE_INPUT_R1.fastq.gz
WT_NAIVE_LEF1_IP_R1.fastq.gz
WT_NAIVE_LEF1_IP_R2.fastq.gz
WT_NAIVE_LEF1_IP_R3.fastq.gz
WT_NAIVE_SOX2_IP_R1.fastq.gz

WT_NAIVE_SOX2_IP_R2.fastq.gz
WT_NAIVE_SOX2_IP_R3.fastq.gz
WT_NAIVE_TCF3_IP_R1.fastq.gz
WT_NAIVE_TCF3_IP_R2.fastq.gz
WT_NAIVE_TCF3_IP_R3.fastq.gz
WT_NAIVE_TCF4_IP_R1.fastq.gz
WT_NAIVE_TCF4_IP_R2.fastq.gz
WT_NAIVE_TCF4_IP_R3.fastq.gz
D3_SOX2_CHIR_FGF_BCATENIN_IP_R1.fastq.gz
D3_SOX2_CHIR_FGF_BCATENIN_IP_R2.fastq.gz
D3_SOX2_CHIR_FGF_BCATENIN_IP_R3.fastq.gz
D3_SOX2_CHIR_FGF_DOX_BCATENIN_IP_R1.fastq.gz
D3_SOX2_CHIR_FGF_DOX_BCATENIN_IP_R2.fastq.gz
D3_SOX2_CHIR_FGF_DOX_BCATENIN_IP_R3.fastq.gz
D3_SOX2_CHIR_FGF_DOX_INPUT_R1.fastq.gz
D3_SOX2_CHIR_FGF_DOX_LEF1_IP_R1.fastq.gz
D3_SOX2_CHIR_FGF_DOX_LEF1_IP_R2.fastq.gz
D3_SOX2_CHIR_FGF_DOX_LEF1_IP_R3.fastq.gz
D3_SOX2_CHIR_FGF_DOX_SOX2_IP_R1.fastq.gz
D3_SOX2_CHIR_FGF_DOX_SOX2_IP_R2.fastq.gz
D3_SOX2_CHIR_FGF_DOX_SOX2_IP_R3.fastq.gz
D3_SOX2_CHIR_FGF_DOX_TCF3_IP_R1.fastq.gz
D3_SOX2_CHIR_FGF_DOX_TCF3_IP_R2.fastq.gz
D3_SOX2_CHIR_FGF_DOX_TCF3_IP_R3.fastq.gz
D3_SOX2_CHIR_FGF_DOX_TCF4_IP_R1.fastq.gz
D3_SOX2_CHIR_FGF_DOX_TCF4_IP_R2.fastq.gz
D3_SOX2_CHIR_FGF_DOX_TCF4_IP_R3.fastq.gz
D3_SOX2_CHIR_FGF_INPUT_R1.fastq.gz
D3_SOX2_CHIR_FGF_LEF1_IP_R1.fastq.gz
D3_SOX2_CHIR_FGF_LEF1_IP_R2.fastq.gz
D3_SOX2_CHIR_FGF_LEF1_IP_R3.fastq.gz
D3_SOX2_CHIR_FGF_SOX2_IP_R1.fastq.gz
D3_SOX2_CHIR_FGF_SOX2_IP_R2.fastq.gz
D3_SOX2_CHIR_FGF_SOX2_IP_R3.fastq.gz
D3_SOX2_CHIR_FGF_TCF3_IP_R1.fastq.gz
D3_SOX2_CHIR_FGF_TCF3_IP_R2.fastq.gz
D3_SOX2_CHIR_FGF_TCF3_IP_R3.fastq.gz
D3_SOX2_CHIR_FGF_TCF4_IP_R1.fastq.gz
D3_SOX2_CHIR_FGF_TCF4_IP_R2.fastq.gz
D3_SOX2_CHIR_FGF_TCF4_IP_R3.fastq.gz
D3_WT_CHIR_FGF_BCATENIN_IP_R1.fastq.gz
D3_WT_CHIR_FGF_BCATENIN_IP_R2.fastq.gz
D3_WT_CHIR_FGF_BCATENIN_IP_R3.fastq.gz
D3_WT_CHIR_FGF_INPUT_R1.fastq.gz
D3_WT_CHIR_FGF_LEF1_IP_R1.fastq.gz
D3_WT_CHIR_FGF_LEF1_IP_R2.fastq.gz
D3_WT_CHIR_FGF_LEF1_IP_R3.fastq.gz
D3_WT_CHIR_FGF_SOX2_IP_R1.fastq.gz
D3_WT_CHIR_FGF_SOX2_IP_R2.fastq.gz
D3_WT_CHIR_FGF_SOX2_IP_R3.fastq.gz
D3_WT_CHIR_FGF_TCF3_IP_R1.fastq.gz
D3_WT_CHIR_FGF_TCF3_IP_R2.fastq.gz
D3_WT_CHIR_FGF_TCF3_IP_R3.fastq.gz
D3_WT_CHIR_FGF_TCF4_IP_R1.fastq.gz
D3_WT_CHIR_FGF_TCF4_IP_R2.fastq.gz
D3_WT_CHIR_FGF_TCF4_IP_R3.fastq.gz
D3_BRA_CHIR_FGF_BCATENIN_IP_R1.fastq.gz
D3_BRA_CHIR_FGF_BCATENIN_IP_R2.fastq.gz
D3_BRA_CHIR_FGF_BCATENIN_IP_R3.fastq.gz
D3_BRA_CHIR_FGF_INPUT_R1.fastq.gz
D3_BRA_CHIR_FGF_INPUT_R2.fastq.gz
D3_BRA_CHIR_FGF_INPUT_R3.fastq.gz
D3_BRA_CHIR_FGF_LEF1_IP_R1.fastq.gz
D3_BRA_CHIR_FGF_LEF1_IP_R2.fastq.gz
D3_BRA_CHIR_FGF_LEF1_IP_R3.fastq.gz
D3_BRA_CHIR_FGF_SOX2_IP_R1.fastq.gz
D3_BRA_CHIR_FGF_SOX2_IP_R2.fastq.gz
D3_BRA_CHIR_FGF_SOX2_IP_R3.fastq.gz
D3_CDX_CHIR_FGF_BCATENIN_IP_R1.fastq.gz
D3_CDX_CHIR_FGF_BCATENIN_IP_R2.fastq.gz
D3_CDX_CHIR_FGF_BCATENIN_IP_R3.fastq.gz
D3_CDX_CHIR_FGF_INPUT_R1.fastq.gz
D3_CDX_CHIR_FGF_INPUT_R2.fastq.gz
D3_CDX_CHIR_FGF_INPUT_R3.fastq.gz
D3_CDX_CHIR_FGF_LEF1_IP_R1.fastq.gz
D3_CDX_CHIR_FGF_LEF1_IP_R2.fastq.gz

D3_CDX_CHIR_FGF_LEF1_IP_R3.fastq.gz
D3_CDX_CHIR_FGF_SOX2_IP_R1.fastq.gz
D3_CDX_CHIR_FGF_SOX2_IP_R2.fastq.gz
D3_CDX_CHIR_FGF_SOX2_IP_R3.fastq.gz
D3_WT_CHIR_FGF_BCATENIN_IP_R1.fastq.gz
D3_WT_CHIR_FGF_BCATENIN_IP_R2.fastq.gz
D3_WT_CHIR_FGF_BCATENIN_IP_R3.fastq.gz
D3_WT_CHIR_FGF_INPUT_R1.fastq.gz
D3_WT_CHIR_FGF_INPUT_R2.fastq.gz
D3_WT_CHIR_FGF_INPUT_R3.fastq.gz
D3_WT_CHIR_FGF_LEF1_IP_R1.fastq.gz
D3_WT_CHIR_FGF_LEF1_IP_R2.fastq.gz
D3_WT_CHIR_FGF_LEF1_IP_R3.fastq.gz
D3_WT_CHIR_FGF_SOX2_IP_R1.fastq.gz
D3_WT_CHIR_FGF_SOX2_IP_R2.fastq.gz
D3_WT_CHIR_FGF_SOX2_IP_R3.fastq.gz
D3_WT_FGF_INPUT_R1.fastq.gz
D3_WT_FGF_INPUT_R2.fastq.gz
D3_WT_FGF_INPUT_R3.fastq.gz
D3_WT_FGF_SOX2_IP_R1.fastq.gz
D3_WT_FGF_SOX2_IP_R2.fastq.gz
D3_WT_FGF_SOX2_IP_R3.fastq.gz
WT_NAIVE_BCATENIN_IP_R1.narrowPeak.gz
WT_NAIVE_BCATENIN_IP_R2.narrowPeak.gz
WT_NAIVE_BCATENIN_IP_R3.narrowPeak.gz
WT_NAIVE_LEF1_IP_R1.narrowPeak.gz
WT_NAIVE_LEF1_IP_R2.narrowPeak.gz
WT_NAIVE_LEF1_IP_R3.narrowPeak.gz
WT_NAIVE_SOX2_IP_R1.narrowPeak.gz
WT_NAIVE_SOX2_IP_R2.narrowPeak.gz
WT_NAIVE_SOX2_IP_R3.narrowPeak.gz
WT_NAIVE_TCF3_IP_R1.narrowPeak.gz
WT_NAIVE_TCF3_IP_R2.narrowPeak.gz
WT_NAIVE_TCF3_IP_R3.narrowPeak.gz
WT_NAIVE_TCF4_IP_R1.narrowPeak.gz
WT_NAIVE_TCF4_IP_R2.narrowPeak.gz
WT_NAIVE_TCF4_IP_R3.narrowPeak.gz
D3_SOX2_CHIR_FGF_BCATENIN_IP_R1.narrowPeak.gz
D3_SOX2_CHIR_FGF_BCATENIN_IP_R2.narrowPeak.gz
D3_SOX2_CHIR_FGF_BCATENIN_IP_R3.narrowPeak.gz
D3_SOX2_CHIR_FGF_DOX_BCATENIN_IP_R1.narrowPeak.gz
D3_SOX2_CHIR_FGF_DOX_BCATENIN_IP_R2.narrowPeak.gz
D3_SOX2_CHIR_FGF_DOX_BCATENIN_IP_R3.narrowPeak.gz
D3_SOX2_CHIR_FGF_DOX_LEF1_IP_R1.narrowPeak.gz
D3_SOX2_CHIR_FGF_DOX_LEF1_IP_R2.narrowPeak.gz
D3_SOX2_CHIR_FGF_DOX_LEF1_IP_R3.narrowPeak.gz
D3_SOX2_CHIR_FGF_DOX_SOX2_IP_R1.narrowPeak.gz
D3_SOX2_CHIR_FGF_DOX_SOX2_IP_R2.narrowPeak.gz
D3_SOX2_CHIR_FGF_DOX_SOX2_IP_R3.narrowPeak.gz
D3_SOX2_CHIR_FGF_DOX_TCF3_IP_R1.narrowPeak.gz
D3_SOX2_CHIR_FGF_DOX_TCF3_IP_R2.narrowPeak.gz
D3_SOX2_CHIR_FGF_DOX_TCF3_IP_R3.narrowPeak.gz
D3_SOX2_CHIR_FGF_DOX_TCF4_IP_R1.narrowPeak.gz
D3_SOX2_CHIR_FGF_DOX_TCF4_IP_R2.narrowPeak.gz
D3_SOX2_CHIR_FGF_DOX_TCF4_IP_R3.narrowPeak.gz
D3_SOX2_CHIR_FGF_LEF1_IP_R1.narrowPeak.gz
D3_SOX2_CHIR_FGF_LEF1_IP_R2.narrowPeak.gz
D3_SOX2_CHIR_FGF_LEF1_IP_R3.narrowPeak.gz
D3_SOX2_CHIR_FGF_SOX2_IP_R1.narrowPeak.gz
D3_SOX2_CHIR_FGF_SOX2_IP_R2.narrowPeak.gz
D3_SOX2_CHIR_FGF_SOX2_IP_R3.narrowPeak.gz
D3_SOX2_CHIR_FGF_TCF3_IP_R1.narrowPeak.gz
D3_SOX2_CHIR_FGF_TCF3_IP_R2.narrowPeak.gz
D3_SOX2_CHIR_FGF_TCF3_IP_R3.narrowPeak.gz
D3_SOX2_CHIR_FGF_TCF4_IP_R1.narrowPeak.gz
D3_SOX2_CHIR_FGF_TCF4_IP_R2.narrowPeak.gz
D3_SOX2_CHIR_FGF_TCF4_IP_R3.narrowPeak.gz
D3_WT_CHIR_FGF_BCATENIN_IP_R1.narrowPeak.gz
D3_WT_CHIR_FGF_BCATENIN_IP_R2.narrowPeak.gz
D3_WT_CHIR_FGF_BCATENIN_IP_R3.narrowPeak.gz
D3_WT_CHIR_FGF_LEF1_IP_R1.narrowPeak.gz
D3_WT_CHIR_FGF_LEF1_IP_R2.narrowPeak.gz
D3_WT_CHIR_FGF_LEF1_IP_R3.narrowPeak.gz
D3_WT_CHIR_FGF_SOX2_IP_R1.narrowPeak.gz
D3_WT_CHIR_FGF_SOX2_IP_R2.narrowPeak.gz
D3_WT_CHIR_FGF_SOX2_IP_R3.narrowPeak.gz

D3_WT_CHIR_FGF_TCF3_IP_R1.narrowPeak.gz
D3_WT_CHIR_FGF_TCF3_IP_R2.narrowPeak.gz
D3_WT_CHIR_FGF_TCF3_IP_R3.narrowPeak.gz
D3_WT_CHIR_FGF_TCF4_IP_R1.narrowPeak.gz
D3_WT_CHIR_FGF_TCF4_IP_R2.narrowPeak.gz
D3_WT_CHIR_FGF_TCF4_IP_R3.narrowPeak.gz
WT_NAIVE_BCATENIN_IP_R1.bigWig
WT_NAIVE_BCATENIN_IP_R2.bigWig
WT_NAIVE_BCATENIN_IP_R3.bigWig
WT_NAIVE_INPUT_R1.bigWig
WT_NAIVE_LEF1_IP_R1.bigWig
WT_NAIVE_LEF1_IP_R2.bigWig
WT_NAIVE_LEF1_IP_R3.bigWig
WT_NAIVE_SOX2_IP_R1.bigWig
WT_NAIVE_SOX2_IP_R2.bigWig
WT_NAIVE_SOX2_IP_R3.bigWig
WT_NAIVE_TCF3_IP_R1.bigWig
WT_NAIVE_TCF3_IP_R2.bigWig
WT_NAIVE_TCF3_IP_R3.bigWig
WT_NAIVE_TCF4_IP_R1.bigWig
WT_NAIVE_TCF4_IP_R2.bigWig
WT_NAIVE_TCF4_IP_R3.bigWig
D3_SOX2_CHIR_FGF_BCATENIN_IP_R1.bigWig
D3_SOX2_CHIR_FGF_BCATENIN_IP_R2.bigWig
D3_SOX2_CHIR_FGF_BCATENIN_IP_R3.bigWig
D3_SOX2_CHIR_FGF_DOX_BCATENIN_IP_R1.bigWig
D3_SOX2_CHIR_FGF_DOX_BCATENIN_IP_R2.bigWig
D3_SOX2_CHIR_FGF_DOX_BCATENIN_IP_R3.bigWig
D3_SOX2_CHIR_FGF_DOX_INPUT_R1.bigWig
D3_SOX2_CHIR_FGF_DOX_LEF1_IP_R1.bigWig
D3_SOX2_CHIR_FGF_DOX_LEF1_IP_R2.bigWig
D3_SOX2_CHIR_FGF_DOX_LEF1_IP_R3.bigWig
D3_SOX2_CHIR_FGF_DOX_SOX2_IP_R1.bigWig
D3_SOX2_CHIR_FGF_DOX_SOX2_IP_R2.bigWig
D3_SOX2_CHIR_FGF_DOX_SOX2_IP_R3.bigWig
D3_SOX2_CHIR_FGF_DOX_TCF3_IP_R1.bigWig
D3_SOX2_CHIR_FGF_DOX_TCF3_IP_R2.bigWig
D3_SOX2_CHIR_FGF_DOX_TCF3_IP_R3.bigWig
D3_SOX2_CHIR_FGF_DOX_TCF4_IP_R1.bigWig
D3_SOX2_CHIR_FGF_DOX_TCF4_IP_R2.bigWig
D3_SOX2_CHIR_FGF_DOX_TCF4_IP_R3.bigWig
D3_SOX2_CHIR_FGF_INPUT_R1.bigWig
D3_SOX2_CHIR_FGF_LEF1_IP_R1.bigWig
D3_SOX2_CHIR_FGF_LEF1_IP_R2.bigWig
D3_SOX2_CHIR_FGF_LEF1_IP_R3.bigWig
D3_SOX2_CHIR_FGF_SOX2_IP_R1.bigWig
D3_SOX2_CHIR_FGF_SOX2_IP_R2.bigWig
D3_SOX2_CHIR_FGF_SOX2_IP_R3.bigWig
D3_SOX2_CHIR_FGF_TCF3_IP_R1.bigWig
D3_SOX2_CHIR_FGF_TCF3_IP_R2.bigWig
D3_SOX2_CHIR_FGF_TCF3_IP_R3.bigWig
D3_SOX2_CHIR_FGF_TCF4_IP_R1.bigWig
D3_SOX2_CHIR_FGF_TCF4_IP_R2.bigWig
D3_SOX2_CHIR_FGF_TCF4_IP_R3.bigWig
D3_WT_CHIR_FGF_BCATENIN_IP_R1.bigWig
D3_WT_CHIR_FGF_BCATENIN_IP_R2.bigWig
D3_WT_CHIR_FGF_BCATENIN_IP_R3.bigWig
D3_WT_CHIR_FGF_INPUT_R1.bigWig
D3_WT_CHIR_FGF_LEF1_IP_R1.bigWig
D3_WT_CHIR_FGF_LEF1_IP_R2.bigWig
D3_WT_CHIR_FGF_LEF1_IP_R3.bigWig
D3_WT_CHIR_FGF_SOX2_IP_R1.bigWig
D3_WT_CHIR_FGF_SOX2_IP_R2.bigWig
D3_WT_CHIR_FGF_SOX2_IP_R3.bigWig
D3_WT_CHIR_FGF_TCF3_IP_R1.bigWig
D3_WT_CHIR_FGF_TCF3_IP_R2.bigWig
D3_WT_CHIR_FGF_TCF3_IP_R3.bigWig
D3_WT_CHIR_FGF_TCF4_IP_R1.bigWig
D3_WT_CHIR_FGF_TCF4_IP_R2.bigWig
D3_WT_CHIR_FGF_TCF4_IP_R3.bigWig
D3_BRA_CHIR_FGF_BCATENIN_IP_R1.narrowPeak.gz
D3_BRA_CHIR_FGF_BCATENIN_IP_R2.narrowPeak.gz
D3_BRA_CHIR_FGF_BCATENIN_IP_R3.narrowPeak.gz
D3_BRA_CHIR_FGF_LEF1_IP_R1.narrowPeak.gz
D3_BRA_CHIR_FGF_LEF1_IP_R2.narrowPeak.gz
D3_BRA_CHIR_FGF_LEF1_IP_R3.narrowPeak.gz

D3_BRA_CHIR_FGF_SOX2_IP_R1.narrowPeak.gz
D3_BRA_CHIR_FGF_SOX2_IP_R2.narrowPeak.gz
D3_BRA_CHIR_FGF_SOX2_IP_R3.narrowPeak.gz
D3_CDX_CHIR_FGF_BCATENIN_IP_R1.narrowPeak.gz
D3_CDX_CHIR_FGF_BCATENIN_IP_R2.narrowPeak.gz
D3_CDX_CHIR_FGF_BCATENIN_IP_R3.narrowPeak.gz
D3_CDX_CHIR_FGF_LEF1_IP_R1.narrowPeak.gz
D3_CDX_CHIR_FGF_LEF1_IP_R2.narrowPeak.gz
D3_CDX_CHIR_FGF_LEF1_IP_R3.narrowPeak.gz
D3_CDX_CHIR_FGF_SOX2_IP_R1.narrowPeak.gz
D3_CDX_CHIR_FGF_SOX2_IP_R2.narrowPeak.gz
D3_CDX_CHIR_FGF_SOX2_IP_R3.narrowPeak.gz
D3_WT_CHIR_FGF_BCATENIN_IP_R1.narrowPeak.gz
D3_WT_CHIR_FGF_BCATENIN_IP_R2.narrowPeak.gz
D3_WT_CHIR_FGF_BCATENIN_IP_R3.narrowPeak.gz
D3_WT_CHIR_FGF_LEF1_IP_R1.narrowPeak.gz
D3_WT_CHIR_FGF_LEF1_IP_R2.narrowPeak.gz
D3_WT_CHIR_FGF_LEF1_IP_R3.narrowPeak.gz
D3_WT_CHIR_FGF_SOX2_IP_R1.narrowPeak.gz
D3_WT_CHIR_FGF_SOX2_IP_R2.narrowPeak.gz
D3_WT_CHIR_FGF_SOX2_IP_R3.narrowPeak.gz
D3_WT_FGF_SOX2_IP_R1.narrowPeak.gz
D3_WT_FGF_SOX2_IP_R2.narrowPeak.gz
D3_WT_FGF_SOX2_IP_R3.narrowPeak.gz
D3_BRA_CHIR_FGF_BCATENIN_IP_R1.bigWig
D3_BRA_CHIR_FGF_BCATENIN_IP_R2.bigWig
D3_BRA_CHIR_FGF_BCATENIN_IP_R3.bigWig
D3_BRA_CHIR_FGF_INPUT_R1.bigWig
D3_BRA_CHIR_FGF_INPUT_R2.bigWig
D3_BRA_CHIR_FGF_INPUT_R3.bigWig
D3_BRA_CHIR_FGF_LEF1_IP_R1.bigWig
D3_BRA_CHIR_FGF_LEF1_IP_R2.bigWig
D3_BRA_CHIR_FGF_LEF1_IP_R3.bigWig
D3_BRA_CHIR_FGF_SOX2_IP_R1.bigWig
D3_BRA_CHIR_FGF_SOX2_IP_R2.bigWig
D3_BRA_CHIR_FGF_SOX2_IP_R3.bigWig
D3_CDX_CHIR_FGF_BCATENIN_IP_R1.bigWig
D3_CDX_CHIR_FGF_BCATENIN_IP_R2.bigWig
D3_CDX_CHIR_FGF_BCATENIN_IP_R3.bigWig
D3_CDX_CHIR_FGF_INPUT_R1.bigWig
D3_CDX_CHIR_FGF_INPUT_R2.bigWig
D3_CDX_CHIR_FGF_INPUT_R3.bigWig
D3_CDX_CHIR_FGF_LEF1_IP_R1.bigWig
D3_CDX_CHIR_FGF_LEF1_IP_R2.bigWig
D3_CDX_CHIR_FGF_LEF1_IP_R3.bigWig
D3_CDX_CHIR_FGF_SOX2_IP_R1.bigWig
D3_CDX_CHIR_FGF_SOX2_IP_R2.bigWig
D3_CDX_CHIR_FGF_SOX2_IP_R3.bigWig
D3_WT_CHIR_FGF_BCATENIN_IP_R1.bigWig
D3_WT_CHIR_FGF_BCATENIN_IP_R2.bigWig
D3_WT_CHIR_FGF_BCATENIN_IP_R3.bigWig
D3_WT_CHIR_FGF_INPUT_R1.bigWig
D3_WT_CHIR_FGF_INPUT_R2.bigWig
D3_WT_CHIR_FGF_INPUT_R3.bigWig
D3_WT_CHIR_FGF_LEF1_IP_R1.bigWig
D3_WT_CHIR_FGF_LEF1_IP_R2.bigWig
D3_WT_CHIR_FGF_LEF1_IP_R3.bigWig
D3_WT_CHIR_FGF_SOX2_IP_R1.bigWig
D3_WT_CHIR_FGF_SOX2_IP_R2.bigWig
D3_WT_CHIR_FGF_SOX2_IP_R3.bigWig
D3_WT_FGF_INPUT_R1.bigWig
D3_WT_FGF_INPUT_R2.bigWig
D3_WT_FGF_INPUT_R3.bigWig
D3_WT_FGF_SOX2_IP_R1.bigWig
D3_WT_FGF_SOX2_IP_R2.bigWig
D3_WT_FGF_SOX2_IP_R3.bigWig

Genome browser session
(e.g. UCSC)

n/a

Methodology

Replicates

Three biological replicates for each experimental condition were collected from independent experiments. Samples from each independent set of experimental samples was immunoprecipitated together. Only peaks common to 2/3 replicates per group were carried forward for consensus peak generation and the subsequent differential analysis.

Sequencing depth	Single-end, 76bp reads were sequenced on the Illumina HiSeq 4000 platform with an average yield of ~30 million reads per sample and ~91% alignment rate.
Antibodies	Goat SOX2 R&D Cat#AF2018 RRID:AB_355110 Lot#KOY0316101 5ug/IP<br>Rabbit B-Catenin Invitrogen Cat#71-2700 RRID:AB_2533982 Lot#SH2565754 5ug/IP<br>Goat TCF3 Santa Cruz Cat#sc-8635 RRID:AB_2199133 Lot#L1415 5ug/IP<br>Goat TCF4 Santa Cruz Cat#sc-8631 RRID:AB_2199826 Lot#K0615 5ug/IP<br>Goat LEF1 Millipore Cat#CS200635 Lot#2816642 3ug/IP
Peak calling parameters	BWA MEM version 0.7.17 was used to map the reads to the mouse mm10 genome assembly with the command: "bwa mem -M <GENOME_INDEX> <INPUT_FASTQ> | samtools view -b -h -F 0x0100 -O BAM -o <OUTPUT_BAM> -"<br>MACS2 version 2.1.2 was used to call peaks with the command: "macs2 callpeak -t <IP_BAM> -c <INPUT_BAM> -f BAM -g 1.87e9 -n <OUTPUT_PREFIX> --keep-dup all"
Data quality	The nf-core/chipseq pipeline generated extensive QC metrics that are collated and rendered in a MultiQC report at the end of the pipeline. Samples were assessed for total number of reads, duplication rate, total number of peaks and FRiP score. As a stringency filter, only peaks common to 2/3 replicates per group were carried forward for consensus peak generation and the subsequent differential analysis.
Software	The nf-core/chipseq pipeline (version 1.1.0; Ewels et al., 2020; https://doi.org/10.5281/zenodo.3529400) written in the Nextflow domain specific language (version 19.10.0; Di Tommaso et al., 2017) was used to perform the primary analysis of the samples in conjunction with Singularity (version 2.6.0; Kurtzer et al., 2017). The command used was "nextflow run nf-core/chipseq --input design.csv --genome mm10 --gtf refseq_genes.gtf --single_end --narrow_peak --min_reps_consensus 2 -profile crick -r 1.1.0"

Flow Cytometry

Plots

Confirm that:

☒ The axis labels state the marker and fluorochrome used (e.g. CD4-FITC).

☒ The axis scales are clearly visible. Include numbers along axes only for bottom left plot of group (a 'group' is an analysis of identical markers).

☒ All plots are contour plots with outliers or pseudocolor plots.

☒ A numerical value for number of cells or percentage (with statistics) is provided.

Methodology

Sample preparation	A detailed protocol for sample preparation is provided in the Methods section.
Instrument	Becton Dickinson (BD) LSR Fortessa
Software	Flow cytometry data was acquired using BD FACSDiva software, and analysed in the R programming environment using the flowCore package.
Cell population abundance	No cell-sorting was performed in this study. Flow cytometry was used for quantification only.
Gating strategy	Gates applied in every experiment: SSC-A/FSC-A gate was used to remove debris and dead cells; FSC-A/FSC-H was used to remove cell doublets

☒ Tick this box to confirm that a figure exemplifying the gating strategy is provided in the Supplementary Information.
